# Dietary supplementation of nucleotides and oligosaccharides in kittens reduces the expression of circulating miR-1-3p, miR-133a-3p, miR-206-3p and miR-383-5p

**DOI:** 10.3389/fvets.2024.1382436

**Published:** 2025-11-06

**Authors:** Fabio Albanese, Matthew Harrison, Michelle J. Farquhar, Lucy J. Holcombe, Jujhar Atwal, Phil Watson, Anna M. Piccinini

**Affiliations:** 1 WALTHAM Petcare Science Institute, Melton Mowbray, Leicestershire, United Kingdom; 2 School of Pharmacy, University of Nottingham, Nottingham, United Kingdom

**Keywords:** microRNA, nucleotide, FOS, XOS, feline, immune, development, biomarker

## Abstract

The immune system of kittens is less efficient at fighting pathogens compared to adult cats with kittens being more susceptible to infections. Increasing evidence shows that dietary interventions can enhance immunity in mammals and modulate the expression of microRNAs (miRNAs) with key immune functions, however research in kittens is limited. Diets that can enhance the ability of a kitten's developing immune system to successfully fight infections, and where effects can be monitored by veterinarians, are highly sought-after. Here, we utilised small RNA sequencing (RNA-Seq) to investigate the effects of an experimental (test) diet containing nucleotides and oligosaccharides on the global expression of circulating miRNAs in 23-week-old kittens (*n* = 9). Furthermore, we determined whether these effects were sustained up to 10 weeks post-supplementation. Kittens fed with the test diet were found to have a lower expression of a specific subset of circulating miRNAs, namely miR-1-3p, miR-133a-3p, miR-206-3p and miR-383-5p, compared to animals fed with a control diet. Notably, this effect persisted 10 weeks post-supplementation. Bioinformatics analysis indicated that these miRNAs target immune-related genes and pathways. As such, they may hold potential as biomarkers to monitor immune performance of kittens and inform the prescription of veterinary diets.

## 1 Introduction

Early life represents a critical stage in the development of the feline immune system, during this time kittens are more susceptible to infections than adult animals due to their inability to mount an effective immune response (1). Dietary interventions have previously shown the potential to enhance immunity in adult cats and dogs (2, 3) however, research in kittens is limited. Additional investigation in kittens is necessary for the formulation of specific diets able to support the development of the immune system, providing animals with a higher level of protection against pathogens during a critical stage of their development.

Recently, a review has shed light on the ability of nutrition to modulate gene expression through regulation of microRNAs (miRNAs) (4); endogenous ~22 nucleotide long non-coding RNAs that post-transcriptionally regulate gene expression (5) by targeting specific mRNAs (6). Since their discovery, the identification of miRNAs as immune-biomarkers in humans has increased because of interest in their ability to regulate several immune mechanisms, including the development, differentiation, and function of immune cells (6–8). Efforts are being made to use them for the early detection of infection (9) and to predict vaccine efficacy following immunisation (10). Therefore, the identification of immune-related miRNAs in kittens where expression is affected by nutrition may help veterinarians monitor immune system development and vaccination response, diagnose defective immune functions, and inform clinical interventions.

In mammals, previous work has demonstrated the ability of specific nutrients and overall nutrition to influence the expression of endogenous miRNAs (11), which in turn can regulate immune pathways. Indeed, studies conducted in cell lines revealed the ability of ingredients such as resveratrol to upregulate the anti-inflammatory miR-693, which decreased the activity of AP-1 in THP-1 cells (12). Additionally, fatty acids inhibited the expression of PTEN in hepatocytes by up-regulating miR-21 synthesis (13). Knockout experiments using cell line models in mice demonstrated the important role that miRNAs play during the maturation of immune cells. In fact, a defective interaction between dendritic cells and T lymphocytes, and the inability to mount acquired immune responses, were observed following miR-155 knockout (14, 15). Moreover, the absence of the RNA endonuclease Dicer caused an impaired development of T-helper (Th) lymphocytes and a reduced proliferation of T cells upon stimulation (16, 17). In kittens, Zhou et al. (18) showed that the expression level of miRNAs in 10-week-old animals was altered following *in vivo* infection with Feline parvovirus (FPV), and that several miRNAs potentially regulated the JAK-STAT pathway, thus affecting the synthesis of cytokines with antiviral effect and growth factors generally critical for immune development and haematopoiesis. Furthermore, besides endogenous miRNAs, growing evidence shows that exogenous miRNAs can regulate gene expression between different species (19). Specifically, mammals can uptake exogenous miRNAs, especially food-derived miRNAs (xeno-miRNAs), which have been detected as functional molecules in the blood and other tissues of the host, following the ingestion of vegetables, cereals and fruits (20–22).

Nucleotides are considered semi-essential nutrients that provide energy carrier molecules such as ATP and GTP for energy use and storage at the cellular level and protein synthesis, respectively (23). Although nucleotides are naturally synthesised in the body, tissues with a rapid turnover such as the intestinal epithelium and the lymphoid tissue utilise the salvage pathway and recover nucleosides and nucleobases from the degradation of RNA and DNA. Moreover, when *de novo* synthesis and salvage pathway do not fulfil the needs of cell nucleotides (e.g. during growth, development and inflammation), cells can utilise nucleotides from exogenous sources, including the diet (24, 25). There is evidence that dietary nucleotides positively support immunity, tissue growth and development in infants (23, 26). Nucleotide supplementation also increases bacterial resistance to several pathogens in mice (27–29), as well as supporting the maturation of murine T helper lymphocytes (30) and enhancing the antibody response following influenza and diphtheria immunisations in infants (31).

Prebiotics are non-digestible dietary carbohydrate fibres contained in cereals, fruits, and vegetables, which have been identified as being able to support the development of the gut microbiota and the synthesis of several metabolites, including short chain fatty acids (SCFA), with health benefits (32). Among several prebiotics that occur in nature, fructo-oligosaccharides (FOS) and xylo-oligosaccharides (XOS) are two well-known prebiotics that have been shown to beneficially affect immunity in mammals, including cats and dogs (33–35).

To date, although there is no evidence for the ability of dietary supplementation of nucleotides to affect the expression of endogenous miRNAs, studies have reported that carbohydrates such as FOS and XOS can influence the expression of miRNAs in mammals by epigenetic alterations (e.g. methylation or acetylation) of the promoter regions of miRNAs (36, 37). Specifically, research conducted in rats showed that dietary supplementation of XOS significantly decreased the expression of miR-192-5p and miR-221-3p in the liver, and this was linked to reduced hepatic inflammation (38). Alleviation of hepatic steatosis was also observed in mice following dietary supplementation of FOS and was attributed to decreased expression of miR-33 in the liver which may have contributed to increased fatty acid oxidation (39), as shown in an independent murine study (40). Moreover, Lim et al. (41) showed that mice fed a diet containing xylobiose, a major component of XOS, displayed decreased expression of miR-33a and increased levels of miR-122a in the liver and that this contributed to a reduced hepatic accumulation of lipids.

The potential of dietary ingredients to modulate the expression of endogenous miRNAs and the impact this could have on the immune system and the physiological response to vaccine challenge, prompted us to investigate the effects of feeding a diet fortified with nucleotides, short chain FOS (scFOS) and XOS (test diet) on circulating plasma miRNAs in 23-week-old kittens. We also determined the longer-term impact of the dietary supplements on the levels of plasma miRNAs by quantifying their levels 2 and 10 weeks after the test diet was withdrawn.

## 2 Materials and methods

### 2.1 Animals and study design

**Figure 1:**
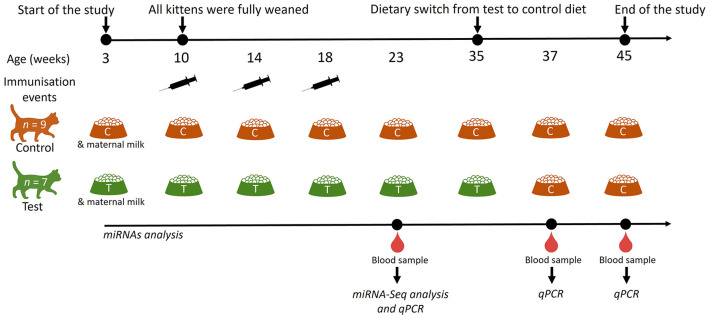
Schematic representation of the study design to determine the effects of dietary nucleotides and oligosaccharides on the expression of circulating miRNAs. Kittens were fed a control (C; *n* = 9) or a test (T; *n* = 7) diet from 3 weeks of age; all kittens were fully weaned at 10 weeks of age. miRNA analysis was performed at 23-, 37- and 45-weeks of age. The test diet group was moved onto a control diet at 35 weeks of age. Cats were vaccinated with Fevaxyn^®^ Pentofel vaccine at 10, 14 and 18 weeks of age.

Domestic short haired kittens used in this study were bred and housed in purpose-built, environmentally enriched housing at the Waltham Petcare Science Institute. All 16 kittens (*n* = 5 females and *n* = 11 males) enrolled in this study were clinically healthy. This work was reviewed and approved by the Waltham Animal Welfare and Ethical Review Board and conducted under the authority of the Animals (Scientific Procedures) Act 1986. In a blinded parallel study, six entire litters of kittens were randomly allocated to either a control (*n* = 9 kittens; *n* = 4 litters) or test (*n* = 7 kittens; *n* = 2 litters) diet group (Figure 1). Blood samples were used to conduct analysis of miRNAs. Kittens were enrolled in this study at 3 weeks of age and fully weaned by 10 weeks. A number of male kittens (three in the control group and one in the test group) were neutered at 29 weeks of age. Following blood collection at 35-weeks of age, test diet kittens were moved onto the control diet until 45 weeks old (Test-Control; T-C kittens), whereas kittens fed with the control diet were maintained on the same diet until 45 weeks of age (Control-Control; C-C kittens). The expression of plasma miRNAs was determined at 23, 37 and 45 weeks.

### 2.2 Vaccinations

Kittens received, as part of a standard veterinary recommended vaccination protocol, a nasal administration of Nobivac Bb^®^ vaccine at 4 weeks of age that contained live attenuated *Bordetella bronchiseptica* bacteria, and a subcutaneous injection of Fevaxyn^®^ Pentofel vaccine (Zoetis UK Ltd.) at 10, 14 and 18 weeks (± 1 week) of age that contained inactivated forms of feline herpesvirus (strain 605), parvovirus (strain CU4), calicivirus (strain 255), leukaemia virus (strain 61E) and *Chlamydophila felis* bacteria (strain Cello).

### 2.3 Diets

The two experimental diets (control and test) used in this study were extruded dry diets formulated and manufactured by Royal Canin (France). Both contained dried poultry protein, animal fat, maize flour, rice, vegetable protein isolate, hydrolysed animal protein, fish oil, soya oil, beet pulp and vegetable fibres. The test diet was also spray coated with 0.33 g yeast extract rich in nucleotides (approximately constituted by 10% GMP, 20% AMP, 40% CMP and 30% UMP; PetMOD, Prosol, Italy), 0.3 g XOS (XOS 35, Longlive, China) and 0.45 g scFOS (Profeed, Tereos, France; degree of polymerization between 3 and 5) per 100 g of diet. The concentration of nucleotides, scFOS and XOS included in the diet were chosen by Royal Canin based on literature, previous (unpublished) *in vitro* and *in vivo* studies performed by Royal Canin on the individual inclusion of the ingredients, toxicological assessment of the raw materials, and adjustments made to take into account loss in processing of the diet and bioavailability of the compounds (42–44).

The post-production analysis of the experimental diets, carried out at Eurofins Ltd (United Kingdom) and reported in Atwal et al. (45) showed that some differences in nutrient composition between the two diets existed and are likely due to the variability of raw ingredients during the manufacturing process and the analysis performed. The diets were nutritionally complete according to the Association of American Feed Control Officials (AAFCO) (43), and complied with nutritional guidelines produced by The European Pet Food Industry (FEDIAF) (44) and the National Research Council (NRC) (46). Each diet was fed to the kittens at a daily energy intake that was appropriate to their age, in accordance with the guidelines for nutrient requirements for cats (47). Amount of food (g) offered, eaten, and refused were also recorded, as reported in our previous study (45).

### 2.4 Blood collection and plasma preparation and storage

All the cats were fasted for at least three and a half hours before blood collection. Lidocaine-prilocaine analgesic cream was applied 45 min before 2 ml blood was drawn from the jugular vein and decanted into K3-EDTA tubes. Within 1 h of sampling, blood samples were centrifuged at 2000 x g at room temperature for 10 min, plasma collected from the upper phase, and further centrifuged at 3000 x g at 4°C for 15 min, to remove additional cellular debris and to minimise the contamination of cell-free nucleic acids by genomic DNA and RNA derived from damaged blood cells. The cleared supernatants were transferred to fresh RNase-free tubes and frozen at −80°C until required.

### 2.5 miRNA-Seq analysis

Plasma samples from all 23-week-old kittens were sent to QIAGEN (Germany) for next-generation sequencing (NGS) of miRNAs. RNA was isolated using the miRNeasy Serum/Plasma Kit (Cat. No. 217184, QIAGEN), according to the manufacturer's instructions. Prior to sequencing, the preparation of the libraries was performed using the QIAseq miRNA Library Kit (QIAGEN) and total RNA was converted into miRNA next-generation sequencing (NGS) libraries. Amplification of libraries was performed, and quality controlled using capillary electrophoresis (Agilent DNA 1000 Chip). Based on the quality of the inserts and their concentrations, the libraries were pooled in equimolar ratios. The library pools were then sequenced on a NextSeq instrument (Illumina^®^) to produce 12 million reads of 1 × 75 base pair fragments per sample. Raw data was then de-multiplexed using the bcl2fastq software (Illumina^®^), which also created FASTQ files for each sample. The expression of miRNAs was quantified using the Quantify miRNA tool in Genomics Workbench (QIAGEN) and normalised to counts per million reads mapped (CPM). The variance of log_2_CPM in expression levels was then used to conduct a principal component analysis (PCA) in QIAGEN CLC Genomics Workbench (Supplementary Figure 1) to identify the relationship between the top 300 plasma miRNAs with the highest variance across all the samples and diets offered to the kittens in this study.

### 2.6 Isolation of RNA from plasma and quantification

Synthetic cel-miR-2-3p and cel-miR-39-3p molecules (Applied Biosystems), used as exogenous RNA references (spike-ins) to monitor the efficiency of the RNA isolation method and the reverse transcription polymerase chain (RT) reaction, respectively, were reconstituted at 25 μM using RNase/DNase-free (no diethyl pyrocarbonate (DEPC)-treated) water and stored at −20°C until needed. To isolate RNA, plasma samples were defrosted on ice, the synthetic (spike-in) cel-miR-2-3p was diluted 1:10,000,000 and added to plasma samples, and RNA was isolated using the miRNeasy Serum/Plasma Advanced Kit (QIAGEN) and quantified using a Bioanalyzer 2100 instrument and Agilent Small RNA kit (Agilent), following manufacturer's instructions.

### 2.7 Synthesis of miRNA-specific cDNAs and qPCR analysis

The entire amount of RNA isolated was used for the synthesis of cDNA molecules, this was performed immediately after the isolation of RNA from plasma using TaqMan™ MicroRNA Reverse Transcription Kit and 5X TaqMan™ miRNA assays (Thermo Fisher Scientific; Supplementary Table 1). The spike-in cel-miR-39-3p (25 μM) was diluted 1:10,000,000. The synthesis of cDNA (RT reaction) was specifically performed for each of the miRNAs determined in this study, thus 10 RT reactions were performed for each RNA sample. Each RT reaction (total volume of 15 μl) was comprised of 0.15 μl of 100 mM deoxyribonucleotide triphosphate (dNTPs), 1 μl of MultiScribe RT enzyme (50 U/μl), 1.5 μl of 10X RT buffer, 0.19 μl of RNase inhibitor (20 U/μl), 3 μl of 5X RT specific primers, 5 μl of 1:5 diluted (in RNase free water) RNA, 1 μl of diluted cel-miR-39-3p and 3.16 μl of RNase-free water. RT reactions were incubated at 16°C for 30 min, then at 42°C for 30 min, and finally at 85°C for 5 min. The synthesised miRNA-specific cDNAs were immediately amplified by quantitative polymerase chain reaction (qPCR) assay.

### 2.8 qPCR

The qPCR reactions were specifically performed for each of the miRNAs determined in this study (Supplementary Table 1), thus one qPCR reaction was performed for each miRNA-specific cDNA sample, using the TaqMan™ Universal polymerase chain reaction (PCR) MasterMix and 20X TaqMan™ miRNA Assays (Thermo Fisher Scientific). Each qPCR reaction (total volume of 10 μl) comprised 0.5 μl of 20X TaqMan™ specific primers, 5 μl of TaqMan™ universal PCR master mix, 2 μl of miRNA-specific cDNA, and 2.5 μl of RNase free water. The qPCR reactions were incubated, using an ABI QuantStudio 7 Flex Real-Time PCR System, at 50°C for 2 min, then at 95°C for 10 min, followed by 40 cycles during which reactions were incubated at 95°C for 15 s and then at 60°C for 1 min. Amplification plots were generated using QuantStudio™ Real-Time PCR Software, and cycle threshold (Ct) values were determined for all the endogenous and exogenous (spike-ins) miRNAs analysed in this study. Let-7c-5p, miR-99a-5p, miR-146b-5p and miR-140-3p, whose expression did not significantly change between dietary groups based on results generated by the miRNA-sequencing analysis, were selected as candidate normaliser miRNAs. NormFinder algorithm was used to determine stability values and the 2^−ΔΔCt^ method (48) to assess the relative concentration of miRNAs in feline plasma. The number of independent experiments, each of which was performed on a different day, was also reported.

### 2.9 Statistical analysis

The data mean ± standard error of the mean (SEM) and statistical analysis were calculated using GraphPad Prism 8 software. Two sets of hypothesis tests were performed, the difference between diet groups at each of the time points and the change from earliest timepoint to each subsequent time point within each diet group. The analysis to compare the means of expression of each plasma miRNA in 23-, 37- and 45-week-old kittens, between test and control groups, was completed performing an unpaired *t*-test. While a one-way ANOVA and Dunnett's multiple comparisons test were performed to compare the expression levels of miRNAs in the control group over time, a mixed-effects analysis and Dunnett's multiple comparisons test were employed to compare the expression levels of miRNAs in the test group (at 23 weeks of age) compared to T-C groups (at 37 and 45 weeks). *P*-values ≤ 0.05 were considered significant.

### 2.10 Bioinformatics analysis

The miRTarBase database (https://mirtarbase.cuhk.edu.cn/) (49) was used to identify experimentally validated targets of the miRNAs of interest, while the DAVID platform (https://david.ncifcrf.gov/tools.jsp) was used to conduct gene ontology (GO) enrichment and Kyoto Encyclopaedia of Genes and Genomes (KEGG) pathway analyses to determine the biological processes, cellular components and molecular functions potentially impacted by the miRNAs analysed in this study. Furthermore, the interactions between miR-1-3p, miR-133a-3p, miR-206-3p and miR-383-5p were investigated by creating a network map using the miRNet platform (https://www.mirnet.ca/) (50).

## 3 Results

### 3.1 Identification of differentially expressed miRNAs in kitten plasma via miRNA-sequencing

**Figure 2:**
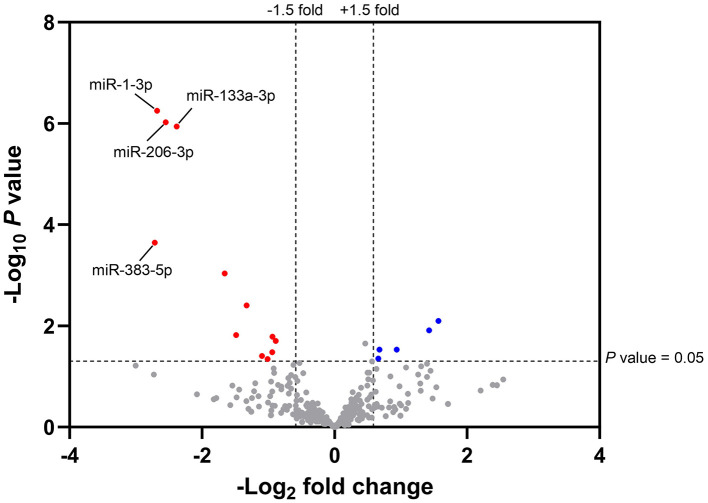
Volcano plot illustrating the magnitude fold change and statistical differences of plasma miRNAs from 23-week-old kittens fed with the test diet compared to control. The magnitude fold change (–Log_2_) is plotted on the *x*-axis and the significance (*t*-test; -Log_10_*P* value) is plotted on the *y*-axis. Fold change > 1.5 and *P*-value < 0.05 were set as the thresholds for identifying significantly differentially expressed plasma miRNAs from 23-week-old kittens fed with the test diet (*n* = 7) compared to the control group (*n* = 9). The red and blue hits represent the downregulated (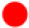) and upregulated (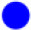) miRNAs in the test group, respectively; the grey hits represent miRNAs that did not meet the threshold criteria (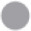). miRNAs ID (*n* = 4) with false discovery rate (FDR) adjusted *P*-value < 0.05 were indicated.

A PCA analysis, did not indicate a clear relationship between plasma miRNAs and diets fed in 23-week-old kittens, although kittens fed with the test diet clustered more than those in the control group. The miRNA sequencing data was further analysed to identify miRNAs with significant differential expression. The relationship between the log_10_ transformed *P-*values and the log_2_ transformed fold change expression values for the test diet compared to the control group are illustrated by volcano plot (Figure 2).

The total number of miRNAs identified in feline plasma was 339. A 1.5-fold change, considered to be sufficient for miRNAs to elicit a biological response, and *P* < 0.05 were set as threshold criteria. In total, 17 miRNAs, 12 of which were downregulated and five upregulated compared to the control group (Supplementary Table 2), met these criteria. To exclude false positive results, *P*-values were adjusted using the Benjamini-Hochberg method (50) and the false discovery rate (FDR) adjusted *P*-values evaluated. This correction identified miR-383-5p, miR-1-3p, miR-133a-3p and miR-206-3p as significantly differentially regulated between dietary groups. These miRNAs, from now referred to as “miRNAs of interest,” had lower expression levels in the plasma of kittens fed with the test diet compared to the control group and were validated by RT-qPCR.

### 3.2 Validation of miRNAs of interest

**Figure 3:**
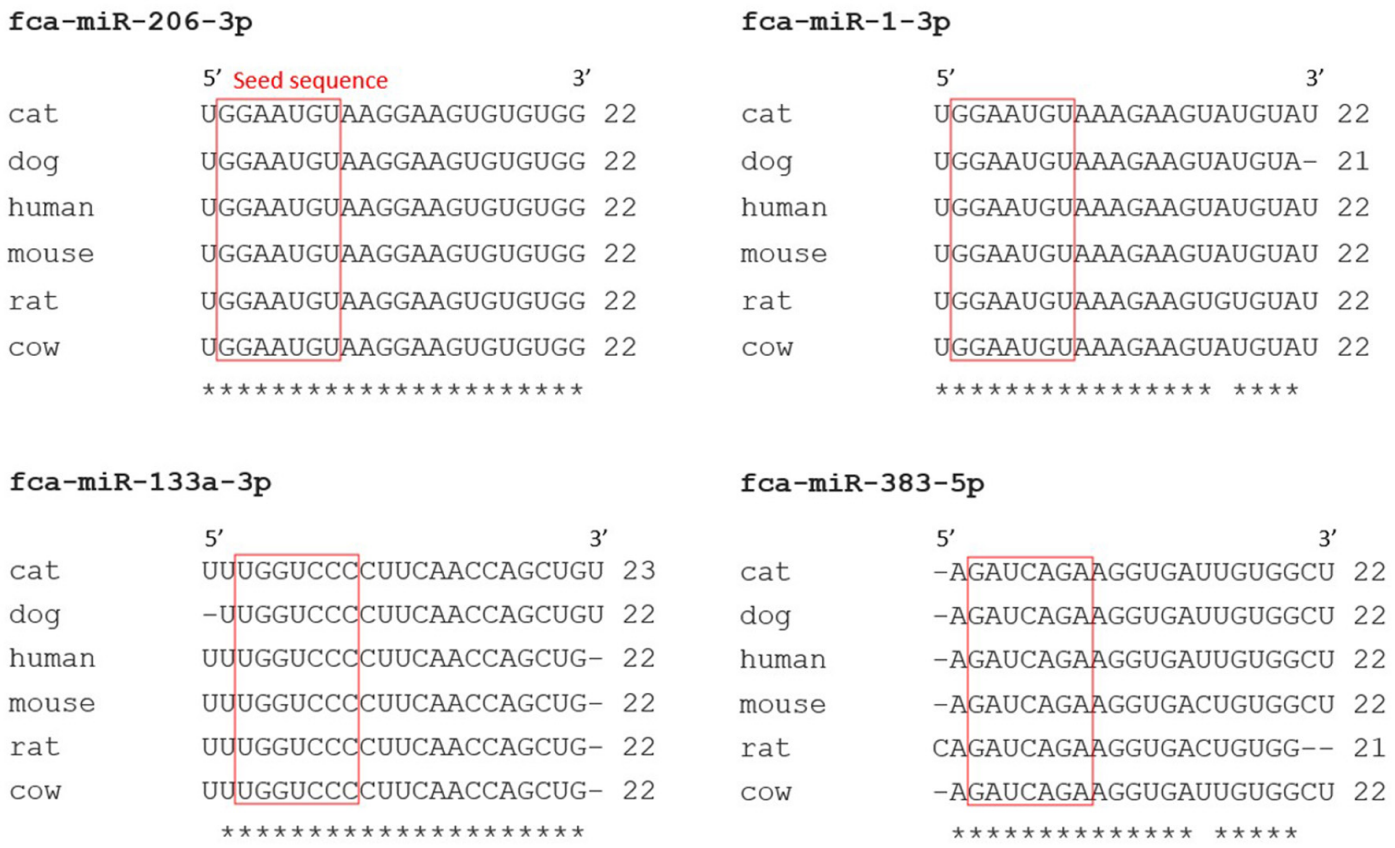
Nucleotide sequences of feline miRNAs with false discovery rate (FDR) <0.05 were well conserved across mammalian species. Nucleotides sequences of feline (*felis catus*; fca) miRNAs of interest obtained from miRNA-sequencing analysis, with FDR <0.05, were aligned (Clustal Omega) with sequences of miRNAs from other species (miRBase). A red box was drawn around miRNA seed regions and similarities between nucleotides across different species were marked with a star (*).

RT-qPCR analysis of the miRNAs of interest was conducted to ensure that these miRNAs were not false positive results of the miRNA-sequencing analysis. Validated feline-specific TaqMan™ miRNA assays were not commercially available, therefore TaqMan™ miRNA assays validated in other species that could be used to amplify feline miRNAs were used. Alignment of the sequences of the feline miRNAs of interest identified in this study to those reported in miRBase for other mammalian species showed 100% homology in the nucleotide sequences for miR-206-3p, and 95%, 91% and 86% homology for miR-133a-3p, miR-1-3p and miR-383-5p, respectively, with a 100% homology in all seed sequences (Figure 3).

To validate the expression of each individual miRNA of interest, miRNA isolation and RT-qPCR experiments were performed on separate plasma aliquots collected from the same kittens analysed by miRNA-sequencing analysis. However, due to limited sample volume, this was only performed in 8 (*n* = 4 for each dietary group) 23-week-old kittens. Gel electrophoresis (Bioanalyzer) confirmed that the RNA isolation from plasma samples retained miRNAs, indicated by the presence of bands in the plasma sample that were similar in size to that of cel-miR-39-3p (22 nucleotides; not shown).

**Figure 4:**
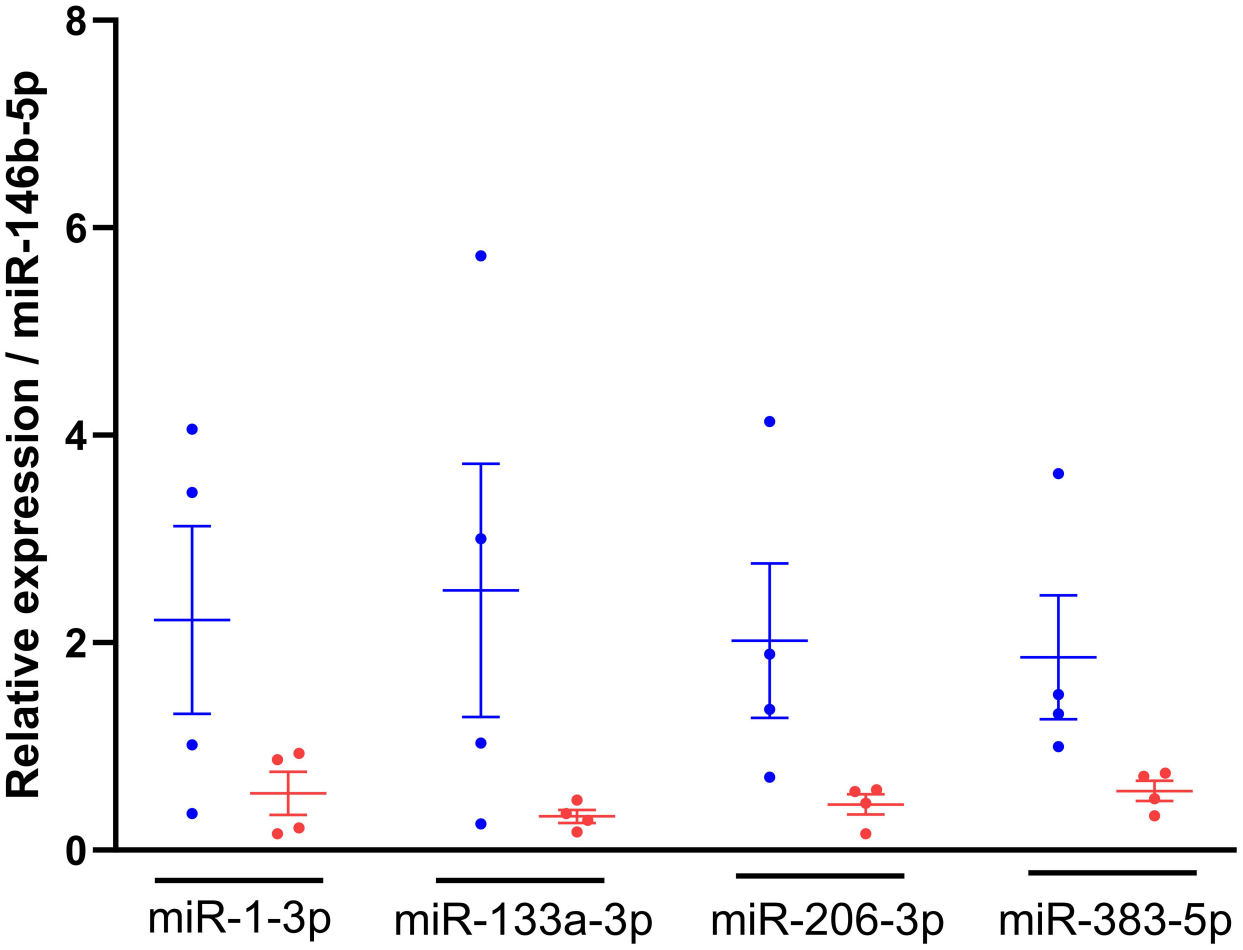
Relative expression of miRNAs of interest was lower in kittens fed the test diet. RNA was isolated from plasma of 23-week-old kittens fed with the control 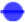 (*n* = 4) or test 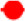 (*n* = 4) diet, miRNA-specific cDNAs were synthesised and TaqMan qPCR performed. The 2^−ΔΔCt^ method was used to quantify the relative expression of miR-1-3p, miR-133a-3p, miR-206-3p and miR-383-5p normalised to miR-146b-5p. Data shown as mean ± SEM (*n* = 6 independent experiments). Unpaired t-test control vs test diet for each miRNA.

RT-qPCR analysis was first performed to identify the most stably expressed miRNA amongst candidate endogenous (let-7c-5p, miR-99a-5p, miR-146b-5p and miR-140-3p) and exogenous (cel-miR-2-3p and cel-miR-39-3p) miRNAs to be used as reference genes for normalisation. As a result, miRNA-146b-5p was selected as normaliser, because it showed a low variability (CV = 1.06%) across all candidate normalisers, did not cluster between diets (Supplementary Figure 2) and had a low stability value (0.005). The relative expression of miR-383-5p, miR-1-3p, miR-133a-3p and miR-206-3p in plasma from 23-week-old kittens (*n* = 4 per diet group) was lower in kittens fed with the test diet compared to the control group, and while their expression was more variable in the control group, the opposite was observed in kittens fed with the test diet (Figure 4).

### 3.3 miRNA target prediction

The targets of feline miRNAs were not present in miRTarBase, however, with the miRNA sequences being highly conserved between cat and human (>86%; Figure 3), the analysis was conducted using the human miRNAs. Validated miRNA-target interactions of human (hsa)-miR-1-3p (miRBase entry MIMAT0000416), hsa-miR-133a-3p (MIMAT0000427), hsa-miR-206-3p (MIMAT0000462) and hsa-miR-383-5p (MIMAT0000738) were identified (Supplementary Table 3). This indicated that a total of 127 genes could be targeted by the miRNAs of interest. Specifically, miR-1-3p, miR-133a-3p, miR-206-3p and miR-383-5p target 78, 39, 31 and 7 genes, respectively, with 28 genes targeted by more than one miRNA.

**Figure 5:**
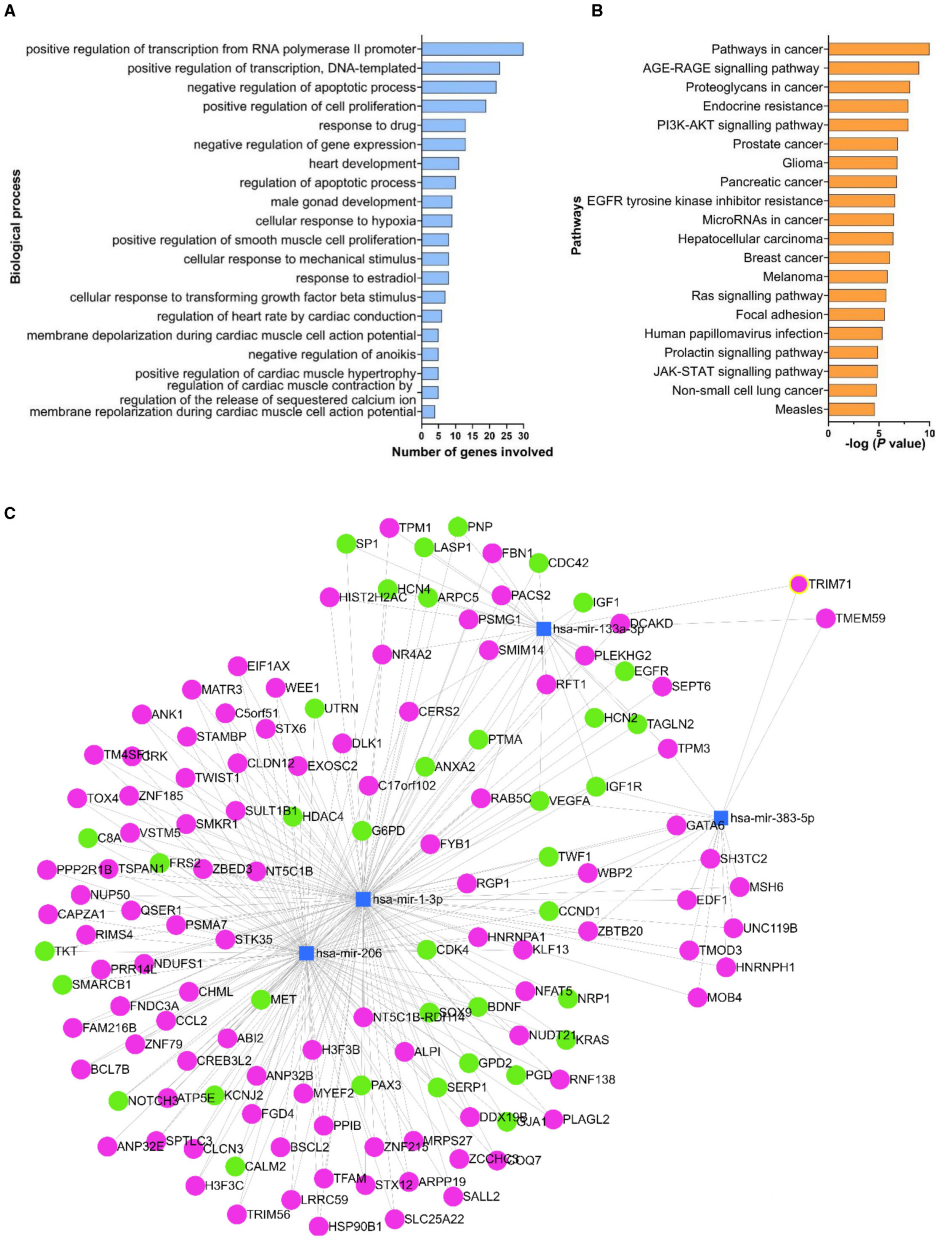
Biological processes, pathways and genes targeted by the miRNAs of interest. The list of experimentally validated genes (identified in miRTarBase) targeted by the miRNAs of interest was used in the DAVID database to determine the biological processes **(A)** and the pathways **(B)** affected by the miRNAs of interest. Only the top-20 statistically significant processes with the lowest *P*-values were reported in **(A)** and **(B)**. Human (hsa)-miR-1-3p, hsa-miR-133a-3p, hsa-miR-206-3p and hsa-miR-383-5p were used to create a network map of the miRNAs of interest (blue squares) using miRNet platform **(C)**. The target genes reported on miRTarBase as being experimentally validated by reporter assay, western blot and qPCR assay were shown in green circles; all the other genes were reported in magenta.

GO enrichment and KEGG pathway analyses, for all 127 validated target genes, identified 352 biological processes, 61 cellular components, and 72 molecular functions, as potentially impacted by the miRNAs of interest. Statistically significant differences were also identified. The top-20 statistically significant biological processes with the lowest *P*-values, together with the number of genes involved in each specific process are reported in Figure 5. The GO analysis showed that the genes targeted by the miRNAs of interest have a role in several biological functions such as positive regulation of transcription and cell proliferation, negative regulation of apoptosis, development and function of cardiac muscle cells (Figure 5A), and molecular processes, including protein and DNA binding (Supplementary Figure 3). The KEGG analysis revealed that the target genes were involved in 114 pathways, and the top-20 statistically significant pathways are reported in Figure 5B and Supplementary Table 4. Additionally, the analysis revealed that the miRNAs of interest could affect different pathways involved in cancer and other diseases, with targeted genes involved in immune mechanisms such as the PI3K-AKT (20 genes; 4.9-fold enrichment) and JAK-STAT (11 genes; 5.9-fold enrichment) pathways.

The network map analysis, conducted using human as reference, indicated that the miRNAs of interest target 1,138 genes in total. Of the 136 genes that were shared between at least two miRNAs of interest, 37 genes were reported on miRTarBase as being experimentally validated by reporter assay, western blot analysis and qPCR assay (Figure 5C). The highest number of common target genes was between miR-1-3p and miR-206-3p, with 96 genes in common, while only one common gene (VEGFA) was targeted by all (*n* = 4) the miRNAs of interest.

### 3.4 Long-term modulation of circulating miRNAs post-supplementation of the test diet

**Figure 6:**
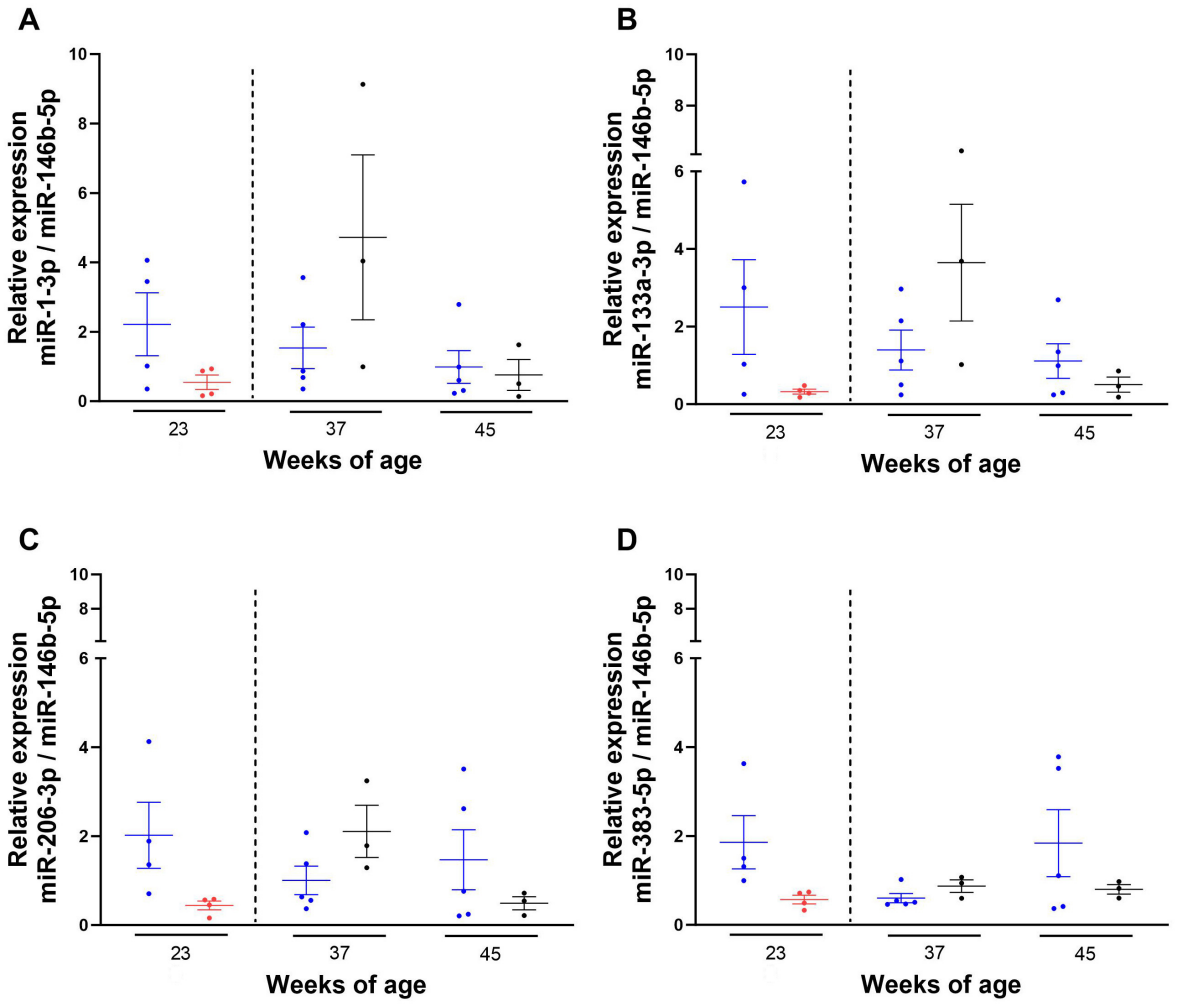
Expression levels of miRNAs of interest 2 and 10 weeks after the withdrawal of the test diet. Kittens 23- (*n* = 4), 37- (*n* = 5) and 45- (*n* = 5) week-old were maintained on the control diet (C-C) 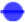 or fed with the test diet 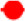 until 23-week-old (*n* = 4), and then moved at 35 weeks (dotted line) onto the control diet 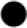 and (blood) sampled at 37 (*n* = 3) and 45 (*n* = 3) weeks of age (T-C). RNA was isolated from plasma of kittens, miRNA-specific cDNAs were synthesised and TaqMan qPCR performed. Ct values obtained for each miRNA were normalised to the averaged Ct value of miR-146b-5p and the 2^−ΔΔCt^ method was used to assess the relative expression of miR-1-3p **(A)**, miR-133a-3p **(B)**, miR-206-3p **(C)** and miR-383-5p **(D)**. Final data shown as mean ± SEM (*n* = 6 independent experiments). Unpaired *t*-test between diets at each week of age. One-way ANOVA and Dunnett's multiple comparisons test vs. 23 weeks of age between control and C-C kittens. Mixed-effects analysis and Dunnett's multiple comparisons test vs. 23 weeks of age between test and T-C groups.

Following quantification of the expression of the miRNAs in plasma from 23-week-old kittens fed with the control or test diet, we also investigated the expression of plasma miRNAs 2 and 10 weeks post-supplementation. Due to limited sample volume, this investigation was conducted on 8 37- and 45-week-old non-matched kittens (*n* = 5 control group; *n* = 3 test group). The expression levels of miRNAs of interest in 37- and 45-week-old kittens were quantified (Figure 6) and compared to miRNA expression data from 23-week-old (non-matched) kittens.

While the expression levels of the miRNAs of interest in 23-week-old kittens were lower in animals fed with the test diet compared to the control group, the opposite was observed at 37 weeks of age, where the expression levels of miR-383-5p, miR-1-3p, miR-133a-3p and miR-206-3p were higher in kittens previously fed with the test diet (T-C kittens) compared to those maintained on the control diet (C-C kittens), with higher variability observed for miR-1-3p and miR-133a-3p (Figure 6).

Conversely, the expression levels of the miRNAs of interest in T-C kittens were lower than the C-C group at 45 weeks of age. None of these changes were statistically significant (*P-*values > 0.5).

## 4. Discussion

In the last decade, nutrition research has revealed the ability of dietary nucleotides and oligosaccharides to support the differentiation and function of immune cells, and the potential for nutrition to affect the expression of endogenous miRNAs in mammals. Therefore, there is a potential for miRNAs to be used as biomarkers to assess the effects of nutrition on immunity, becoming a health monitoring tool to ensure an adequate nutritional support to kittens, during their development. Previously, we found that a diet fortified with nucleotides, scFOS and XOS significantly increased antibody levels post immunisation in kittens, potentially due to an increased polarisation of T-helper cells, activators of antibody-producing B-cells (45). Here, we wanted to determine whether the same diet was also able to affect the expression level of circulating immune-related miRNAs, and if any effects were maintained post-supplementation.

Data generated through the miRNA-sequencing analysis showed a significant difference in miRNA expression between experimental diets, RT-qPCR data was in agreement with this revealing a lower expression of circulating miR-383-5p, miR-1-3p, miR-133a-3p and miR-206-3p in 23-week-old kittens fed with the test diet, compared to control, however this effect was not significant. This may be attributed to differences in sample size between the methods. Indeed, the retrospective powering analysis (not shown) performed by simulation using RT-qPCR data generated in this study, confirmed that 12 kittens were required for 80% power for an effect size of 70%−90%. Unfortunately, we could not recruit additional animals, as this was an opportunistic investigation utilising kittens on an existing study. RT-qPCR data also indicated that the expression levels of the miRNAs of interest were highly variable in kittens fed with the control diet compared to the test group. Although this variability was confirmed by the PCA analysis, further investigation should be conducted as its reason is unclear.

In accordance with previous studies reporting low amounts of cell-free RNA molecules in human plasma, the RNA yield was low in kitten plasma (51, 52). For this reason, the entire amount of unquantified RNA isolated in this study was used for the synthesis of miRNA-specific cDNAs for all the kittens, hence the input total RNA could not be standardised across samples. Nevertheless, Huggett et al. (53) demonstrated that reference genes such as the endogenous miRNA (miR-146b-5p) used in this study can control for different input RNA amounts used in the reverse transcription step.

In animals, miRNAs can regulate more than one gene (54) therefore, miRNAs that usually function in a specific tissue can also regulate the expression of genes in different body compartments. For example, miR-1-3p, miR-133a-3p and miR-206-3p are part of the “myomiRs” family and affect genes involved in skeletal and cardiac muscle development in mammals (55); an association that was confirmed by the GO and KEGG analyses we reported in this study. In addition, those miRNAs can also regulate Fas apoptosis inhibitory molecule (FAIM), an anti-apoptotic protein found in most cell types, including immune cells (56), and affect genes in murine mammary tissue (57) which have shown to affect immunity by regulating NF-κB-mediated inflammatory responses (58), NK cell-mediated killing (59), and the formation of NLRP6 inflammasomes as well as the secretion of IL-18 (60) in humans.

Literature providing evidence of the ability of nutrition to affect the expression of miRNAs in developing kittens has been lacking, potentially due to the low number of miRNA studies conducted in the cat. However, we have revealed for the first time the ability of dietary nucleotides and oligosaccharides to affect potentially immune-related miRNAs in kittens. Indeed, the GO and KEGG analyses we conducted here, indicated that the miRNAs of interest positively regulate cell proliferation and play a role in the PI3K-AKT pathway, potentially supporting the development of immune cells and increasing antibody titres following immunisation. Results suggest that in 23-week-old kittens fed with the test diet, in which a lower expression level of miR-206-3p was measured compared to the control group, the PI3K-AKT pathway was not inhibited. Our hypothesis is supported by previous results generated in a murine preadipocyte cell line, where the expression of miR-206 inhibited c-Met, an important upstream regulator of the PI3K-AKT pathway (61) that is encoded by the MET gene (62), identified here as a validated target of miR-206-3p. The low expression level of miR-206-3p measured in kittens in the test group may have led to sustained PI3K-AKT signalling, which, in turn, may have played a role in supporting the development of T cells and differentiation of T helper lymphocytes in the thymus of kittens. In fact, while the inhibition of the PI3K-AKT pathway in mice led to a reduced number of peripheral T cells (63), its activation was able to induce the differentiation of T cells (64). Moreover, our hypothesis that low miR-206-3p expression may have contributed to the development of T helper lymphocytes in 23-week-old kittens fed with the test diet agrees with data reported in our previous study, where the number of T helper cells observed in kittens fed with the same test diet was higher than that in kittens fed with the control diet, and possibly increased the seroconversion of kittens following vaccination (45).

Here, we determined a lower expression level of miR-1-3p and miR-133a-3p in the test group compared to control. Although there is no evidence showing that these miRNAs function in antibody production post-immunisation and development of T helper lymphocytes, they have been previously studied in the context of immune function. Specifically, research conducted using a murine macrophage cell line (RAW264.7) revealed that miR-1-3p suppressed the phagocytosis of *E. coli* by inhibiting the expression of clathrin heavy chain 1 (CLTC1) gene (65), while miR-133a-3p was able to regulate inflammasome activation through uncoupling protein-2 (UCP2) in a human monocytic cell line (THP1) (66). While UCP2 gene was identified by us as a target gene of miR-133a-3p, our analysis did not report CLTC1 as a validated target of miR-1-3p. Therefore, further investigation is needed to determine and confirm the validated targets of miR-1-3p and miR-133a-3p in the cat and their potential to affect immune functions and pathways.

The expression level of miR-383-5p in plasma of kittens fed with the test diet was also lower compared to the control group. Currently, there is no evidence showing the ability of *in vivo* dietary supplementations to affect the expression level of miR-383-5p, which has mainly been investigated in the context of cancer and identified as a tumour suppressor able to inhibit cell proliferation and cancer progression in several types of cancer (67). Although further investigation is needed to determine the ability of miR-383-5p to influence immune functions, a study conducted by Zhang et al., in the context of the human central nervous system (68) showed that one of the validated targets of miR-383-5p, glycoprotein 130 (gp130), which is the subunit for the receptor of IL-6 family cytokines, induces the activation of the JAK-STAT pathway (69), and its expression is not limited to the nervous system but also found in immune cells (70, 71).

The present study is the first investigating the sustained effects of a diet after its withdrawal on the expression of endogenous miRNAs in kittens. We found that, due to a potential adaptation to the new diet 2 weeks post-supplementation, the expression levels of miR-383-5p, miR-1-3p, miR-133a-3p and miR-206-3p in T-C kittens trended towards being higher than those measured in animals maintained on the control diet (C-C). On the other hand, 10 weeks post-supplementation, the expression levels of these miRNAs were comparable to their levels before the withdrawal of the test diet. One hypothesis is that the effects induced by the test diet on the expression of endogenous miRNAs persisted 10 weeks post-supplementation. Interestingly, on a similar note, a study conducted in piglets showed that the expression levels of miR-708-5p, miR-135-3p and miR-27b-3p were lower in 21-, 35- and 51-day-old piglets previously fed (from 2 to 21 days of age) with a dairy-based formula, compared to animals fed with human breastmilk (72).

The data from this study raise the question whether miR-383-5p, miR-1-3p, miR-133a-3p and miR-206-3p can affect immune functions in kittens. To answer this question, it is imperative to first validate the gene targets of the feline miRNAs of interest that we have identified. This could be achieved through bioinformatics analysis using human miRNAs as references, based on the fact that 90% of the genes in the cat are similar to humans (73). Further future studies incorporating each ingredient individually into the diet, rather than in a cocktail as in the present study, should also be conducted to determine whether the differences observed in expression of miRNAs are driven by single ingredients or their combination.

In summary, our study has found that kittens fed with a diet containing nucleotides, scFOS and XOS express lower levels of circulatory plasma miR-383-5p, miR-1-3p, miR-133a-3p and miR-206-3p compared to the control group; an expression profile that was maintained 10 weeks post-supplementation. The literature and our pathway analysis indicate that these miRNAs regulate the PI3K-AKT and JAK-STAT pathways, suggesting that their modulation may affect the animal immune response, although this was not confirmed.

Finally, in light of our previous work (45), the expression level of the circulating plasma miRNAs identified here may support the development of T cells and increase the seroconversion of kittens following vaccination. However, this cannot be confirmed. Ultimately, our findings identified nutrients able to affect potential immune-related miRNAs, the expression of which could be used to monitor feline immune performance in the future and support veterinary care to improve the healthspan of companion animals.

## Data Availability

The original contributions presented in the study are publicly available. This data can be found here: NCBI repository, PRJNA1334322.

## Supplementary material

Supplementary Figure 1 Principal component analysis (PCA) does not show relationships between plasma miRNAs and diets offered to 23-week-old kittens. Data from plasma of kittens fed with test (*n* = 7) or control (*n* = 9) diet. Expression data was normalised to the counts per million reads mapped (CPM) and variance of the log_2_CPM in expression levels was calculated across all samples. The top 300 miRNAs with the highest variance across all samples were used in the principal component calculation and as input for the PCA plot.

Supplementary Figure 2 MiR-146b was the most suitable miRNA to be used as reference gene in RT-qPCR experiments. Cycle threshold (Ct) values determined by two-step RT-qPCR for individual miRNAs were averaged across 23-week-old kittens fed with the control [[Inline Image]] (*n* = 4) and test [[Inline Image]] (*n* = 4) diets. The variation of individual Ct values from the averaged Ct were calculated together with the coefficient of variation (%CV). Data shown as means ± SEM (*n* = 6 independent experiments).

Supplementary Figure 3 Molecular processes targeted by the miRNAs of interest. The list of experimentally validated genes (identified in miRTarBase) targeted by the miRNAs of interest was used in the DAVID database to determine the molecular processes affected by the miRNAs of interest. Only the top-20 statistically significant processes with the lowest *P*-values were reported.

Supplementary Table 1 Target sequences of TaqMan™ miRNA Assays used in qPCR experiments.

Supplementary Table 2 List of miRNAs that met the set threshold criteria of 1.5-fold change and *P* < 0.05 compared to the control group.

Supplementary Table 3 Experimentally validated miRNA-target of miRNAs of interest reported in miRTarBase database.

Supplementary Table 4 The miRNAs of interest target genes involved in immune signalling pathways.
