# Supplementary figures and images for: Dietary supplementation of nucleotides and oligosaccharides in kittens reduces the expression of circulating miR-1-3p, miR-133a-3p, miR-206-3p and miR-383-5p
Source: Front Vet Sci. 2025 Nov 6;11:1382436. doi: 10.3389/fvets.2024.1382436 (PMC12632807; doi:10.3389/fvets.2024.1382436)

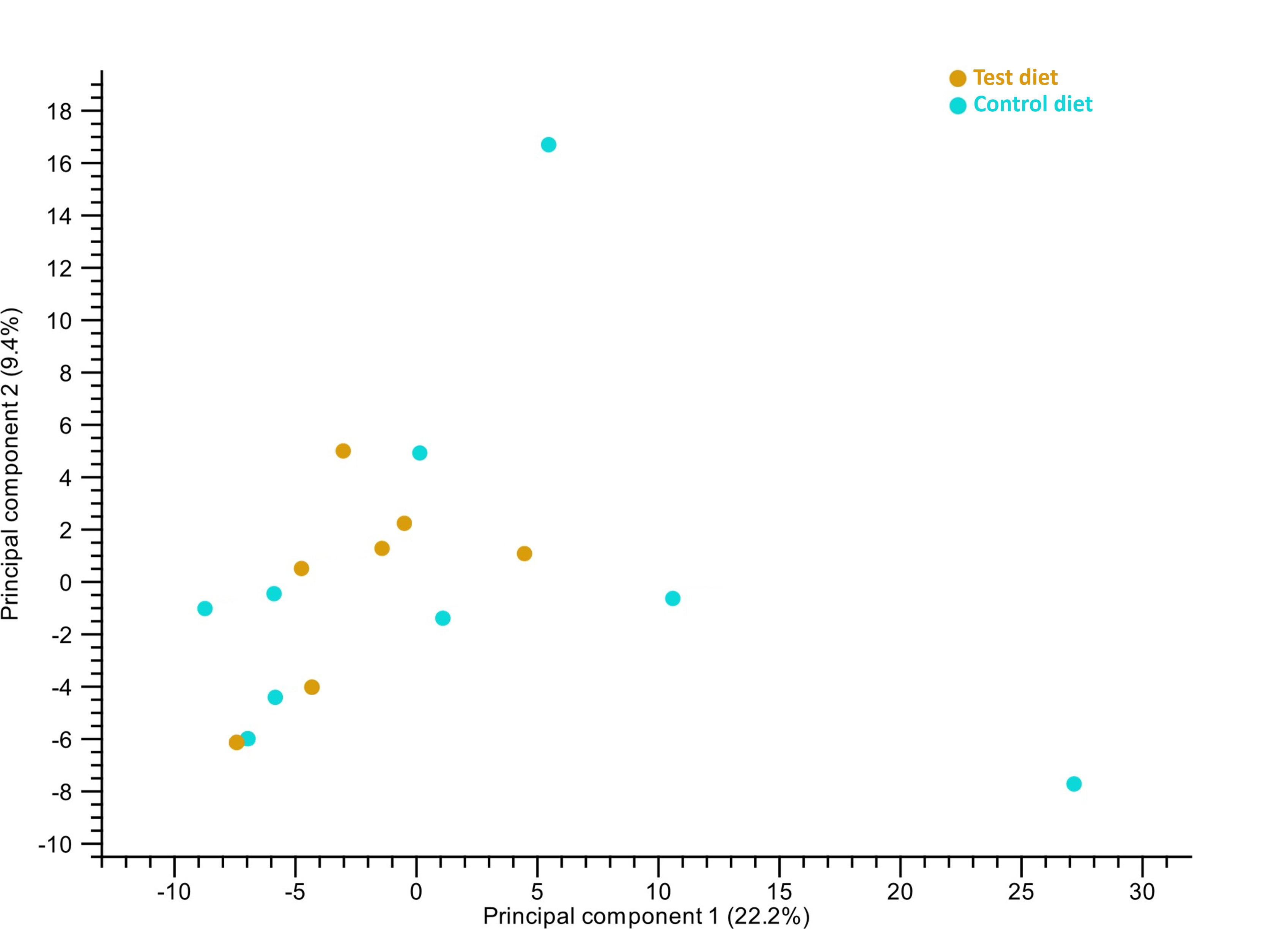

Supplement: Supplementary Figure 1 — Principal component analysis (PCA) does not show relationships between plasma miRNAs and diets offered to 23-week-old kittens. Data from plasma of kittens fed with test (n = 7) or control (n = 9) diet. Expression data was normalised to the counts per million reads mapped (CPM) and variance of the log2CPM in expression levels was calculated across all samples. The top 300 miRNAs with the highest variance across all samples were used in the principal component calculation and as input for the PCA plot. [file Image_1.jpeg]

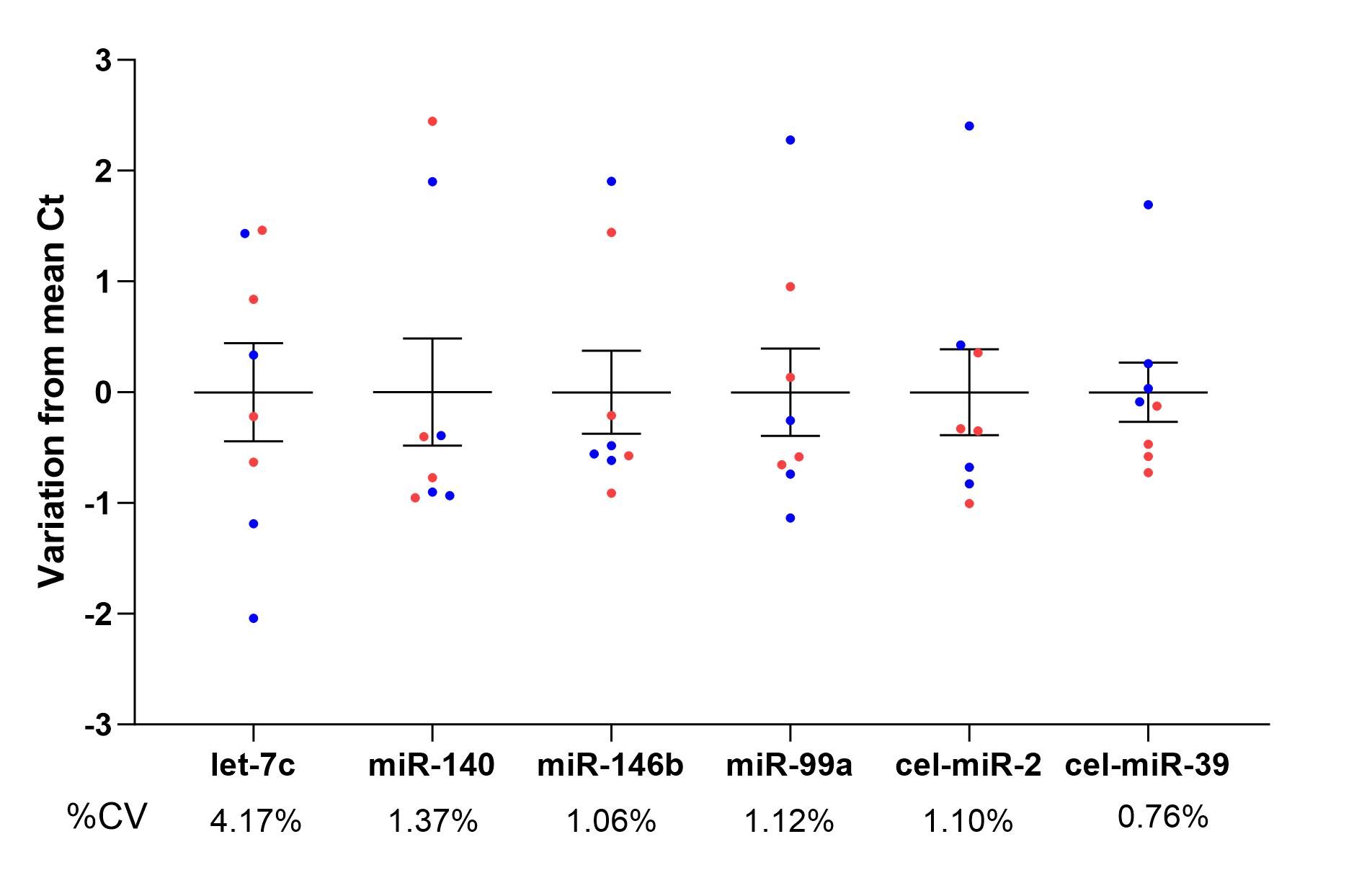

Supplement: Supplementary Figure 2 — MiR-146b was the most suitable miRNA to be used as reference gene in RT-qPCR experiments. Cycle threshold (Ct) values determined by two-step RT-qPCR for individual miRNAs were averaged across 23-week-old kittens fed with the control [[Inline Image]] (n = 4) and test [[Inline Image]] (n = 4) diets. The variation of individual Ct values from the averaged Ct were calculated together with the coefficient of variation (%CV). Data shown as means ± SEM (n = 6 independent experiments). [file Image_2.jpeg]

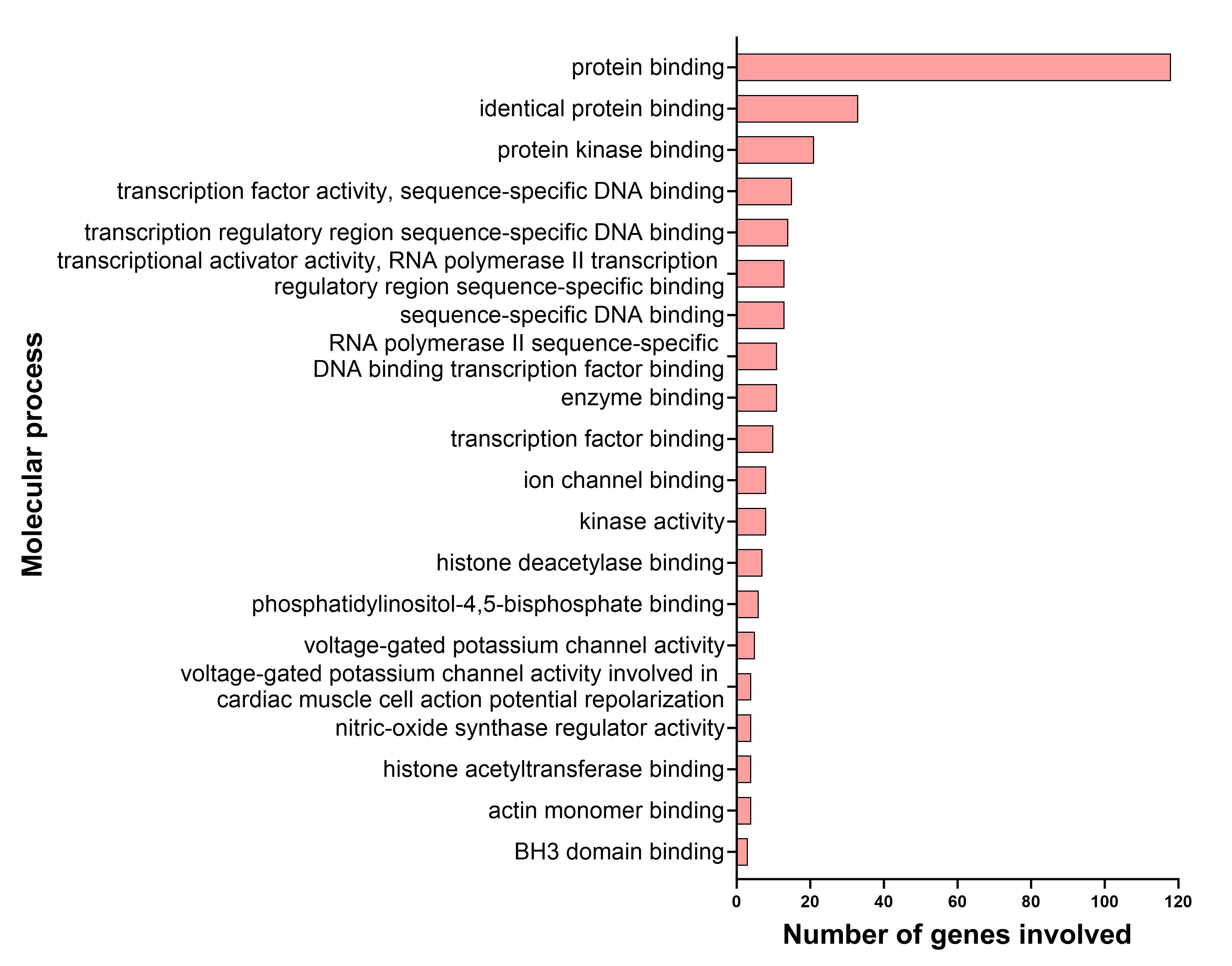

Supplement: Supplementary Figure 3 — Molecular processes targeted by the miRNAs of interest. The list of experimentally validated genes (identified in miRTarBase) targeted by the miRNAs of interest was used in the DAVID database to determine the molecular processes affected by the miRNAs of interest. Only the top-20 statistically significant processes with the lowest P-values were reported. [file Image_3.jpeg]
