# Supplementary material for: Dietary supplementation of nucleotides and oligosaccharides in kittens reduces the expression of circulating miR-1-3p, miR-133a-3p, miR-206-3p and miR-383-5p
Source: Front Vet Sci. 2025 Nov 6;11:1382436. doi: 10.3389/fvets.2024.1382436 (PMC12632807; doi:10.3389/fvets.2024.1382436)
Supplement: Supplementary Table 1 — Target sequences of TaqMan™ miRNA Assays used in qPCR experiments. [file Table_1.docx]

**Supplementary Table 1** – **Target sequences of TaqMan™ miRNA Assays used in qPCR experiments.**

Names, IDs and target sequences of human (hsa), rabbit (ocu), rat (rno), pig (ssc) and *Caenorhabditis elegans* (cel) TaqMan™ miRNA Assays used to amplify miRNAs in quantitative polymerase chain reaction (qPCR) experiments.

|  |  |  | | |  |  | |  |
| --- | --- | --- | --- | --- | --- | --- | --- | --- |
| **Assay name** |  | **Assay ID** |  | **Target sequence** | | |  |  |
|  |  |  | | |  |  | |  |
| hsa-miR-1-3p |  | 2222 |  | UGGAAUGUAAAGAAGUAUGUAU | | |  |  |
| hsa-miR-206-3p |  | 510 |  | UGGAAUGUAAGGAAGUGUGUGG | | |  |  |
| ocu-miR-133a-3p |  | 473241 |  | UUUGGUCCCCUUCAACCAGCUGU | | |  |  |
| hsa-miR-383-5p |  | 573 |  | AGAUCAGAAGGUGAUUGUGGCU | | |  |  |
| hsa-miR-99a-5p |  | 435 |  | AACCCGUAGAUCCGAUCUUGUG | | |  |  |
| hsa-let-7c-5p |  | 379 |  | UGAGGUAGUAGGUUGUAUGGUU | | |  |  |
| rno-miR-146b-5p |  | 2755 |  | UGAGAACUGAAUUCCAUAGGCUGU | | |  |  |
| ssc-miR-140-3p |  | 5800 |  | UACCACAGGGUAGAACCACGGAC | | |  |  |
| cel-miR-2-3p |  | 195 |  | UAUCACAGCCAGCUUUGAUGUGC | | |  |  |
| cel-miR-39-3p |  | 200 |  | UCACCGGGUGUAAAUCAGCUUG | | |  |  |
|  |  |  |  |  |  |  | |  |
