# Supplementary material for: Dietary supplementation of nucleotides and oligosaccharides in kittens reduces the expression of circulating miR-1-3p, miR-133a-3p, miR-206-3p and miR-383-5p
Source: Front Vet Sci. 2025 Nov 6;11:1382436. doi: 10.3389/fvets.2024.1382436 (PMC12632807; doi:10.3389/fvets.2024.1382436)
Supplement: Supplementary Table 2 — List of miRNAs that met the set threshold criteria of 1.5-fold change and P < 0.05 compared to the control group. [file Table_2.docx]

**Supplementary Table 2** - **List of miRNAs that met the set threshold criteria of 1.5-fold change and *P*<0.05 compared to the control group.**Of the 339 plasma feline (*feliscatus*; fca) miRNAs identified in kittens fed with the test (*n* = 7) or control (*n* = 9) diet, only the expression levels of the 17 miRNAs reported here were significantly different between dietary groups; 4 miRNAshad a significant false discovery rate (FDR) adjusted *P* value. *p<0.05.

|  |  |  |  |  |  |  |
| --- | --- | --- | --- | --- | --- | --- |
|  |  | **Control VS Test diet** | | | | |
| **miRNA ID** |  | **Fold change** |  | ***P* value** |  | **FDR *P* value** |
| fca-let-7f-3p |  | 2.961 |  | 0.008 |  | 0.386 |
| fca-miR-483-3p |  | 2.686 |  | 0.012 |  | 0.517 |
| fca-miR-196a-5p |  | 1.914 |  | 0.030 |  | 0.667 |
| fca-miR-26b-5p |  | 1.598 |  | 0.029 |  | 0.667 |
| fca-miR-1249-3p |  | 1.579 |  | 0.044 |  | 0.699 |
| fca-miR-199a-5p |  | -1.850 |  | 0.020 |  | 0.610 |
| fca-miR-99b-5p |  | -1.917 |  | 0.016 |  | 0.557 |
| fca-miR-151-5p |  | -1.921 |  | 0.033 |  | 0.667 |
| fca-miR-1307-3p |  | -2.017 |  | 0.045 |  | 0.699 |
| fca-miR-183-5p |  | -2.137 |  | 0.039 |  | 0.667 |
| fca-miR-143-5p |  | -2.511 |  | 0.004 |  | 0.222 |
| fca-miR-199b-5p |  | -2.804 |  | 0.015 |  | 0.557 |
| fca-miR-326-3p |  | -3.158 |  | 0.001 |  | 0.062 |
| fca-miR-206-3p |  | -5.223 |  | <0.001 |  | <0.001* |
| fca-miR-1-3p |  | -5.846 |  | <0.001 |  | <0.001* |
| fca-miR-133a-3p |  | -6.404 |  | <0.001 |  | <0.001* |
| fca-miR-383-5p |  | -6.569 |  | <0.001 |  | 0.019* |
|  |  |  |  |  |  |  |
