# Supplementary material for: Dietary supplementation of nucleotides and oligosaccharides in kittens reduces the expression of circulating miR-1-3p, miR-133a-3p, miR-206-3p and miR-383-5p
Source: Front Vet Sci. 2025 Nov 6;11:1382436. doi: 10.3389/fvets.2024.1382436 (PMC12632807; doi:10.3389/fvets.2024.1382436)
Supplement: Supplementary Table 3 — Experimentally validated miRNA-target of miRNAs of interest reported in miRTarBase database. [file Table_3.docx]

**Supplementary Table 3 - Experimentally validated miRNA-target of miRNAs of interest reported in miRTarBase database.** Human (hsa)-miR-1-3p, hsa-miR-133a-3p, hsa-miR-206-3p and hsa-miR-383-5p, whose nucleotide sequences were highly conserved with cat miRNAs (Figure 5), were utilised as input sequences on miRTarBase database to identify experimentally validated (by reporter assay, western blot and qPCR assay) target genes of miRNAs of interest investigated in this study. MiRTarBase database entry was also reported.

|  |  |  |  |  |  |  |  |  |  |
| --- | --- | --- | --- | --- | --- | --- | --- | --- | --- |
| **miRNA ID  (hsa)** | **Human target gene** |  | **Reporter  assay** |  | **Western  blot** |  | **qPCR assay** |  | **miRTarBase entry** |
| miR-1-3p | CEBPA |  | ✓ |  | - |  | - |  | MIRT000390 |
|  | MEF2A |  | ✓ |  | - |  | ✓ |  | MIRT000391 |
|  | GATA4 |  | ✓ |  | - |  | - |  | MIRT000393 |
|  | HCN4 |  | ✓ |  | ✓ |  | ✓ |  | MIRT000933 |
|  | HDAC4 |  | ✓ |  | ✓ |  | ✓ |  | MIRT001053 |
|  | FOXP1 |  | ✓ |  | ✓ |  | ✓ |  | MIRT001054 |
|  | HCN2 |  | ✓ |  | ✓ |  | ✓ |  | MIRT001205 |
|  | PTMA |  | ✓ |  | ✓ |  | ✓ |  | MIRT001343 |
|  | MET |  | ✓ |  | ✓ |  | ✓ |  | MIRT001357 |
|  | CAND1 |  | ✓ |  | - |  | - |  | MIRT001378 |
|  | ANXA2 |  | - |  | ✓ |  | ✓ |  | MIRT001388 |
|  | HAND2 |  | ✓ |  | - |  | ✓ |  | MIRT001843 |
|  | IGF1 |  | ✓ |  | ✓ |  | ✓ |  | MIRT001844 |
|  | TMSB4X |  | ✓ |  | - |  | - |  | MIRT001845 |
|  | KCNJ2 |  | ✓ |  | - |  | ✓ |  | MIRT001983 |
|  | GJA1 |  | ✓ |  | ✓ |  | ✓ |  | MIRT001984 |
|  | FN1 |  | ✓ |  | ✓ |  | ✓ |  | MIRT006132 |
|  | XPO6 |  | ✓ |  | ✓ |  | ✓ |  | MIRT002751 |
|  | POGK |  | ✓ |  | - |  | - |  | MIRT002759 |
|  | PGM2 |  | ✓ |  | - |  | - |  | MIRT002766 |
|  | TAGLN2 |  | ✓ |  | ✓ |  | ✓ |  | MIRT002788 |
|  | SERP1 |  | ✓ |  | - |  | - |  | MIRT002806 |
|  | LASP1 |  | ✓ |  | ✓ |  | ✓ |  | MIRT002808 |
|  | NETO2 |  | ✓ |  | - |  | - |  | MIRT002815 |
|  | ADAR |  | ✓ |  | - |  | - |  | MIRT002820 |
|  | KCNE1 |  | ✓ |  | ✓ |  | ✓ |  | MIRT002924 |
|  | BDNF |  | ✓ |  | - |  | - |  | MIRT002955 |
|  | G6PD |  | ✓ |  | ✓ |  | ✓ |  | MIRT002956 |
|  | SOX6 |  | ✓ |  | - |  | - |  | MIRT003203 |
|  | NOTCH3 |  | ✓ |  | ✓ |  | ✓ |  | MIRT006550 |
|  | ATP6V1B2 |  | ✓ |  | - |  | - |  | MIRT003524 |
|  | LARP4 |  | ✓ |  | - |  | - |  | MIRT003525 |
|  | CNN3 |  | ✓ |  | - |  | - |  | MIRT003526 |
|  | PNP |  | ✓ |  | ✓ |  | ✓ |  | MIRT003765 |
|  | SRXN1 |  | ✓ |  | - |  | - |  | MIRT003772 |
|  | KIF2A |  | ✓ |  | - |  | - |  | MIRT003853 |
|  | HSPD1 |  | ✓ |  | - |  | - |  | MIRT003976 |
|  | HSPA4 |  | ✓ |  | - |  | - |  | MIRT003977 |
|  | PIM1 |  | ✓ |  | ✓ |  | ✓ |  | MIRT004322 |
|  | CALM3 |  | ✓ |  | - |  | - |  | MIRT004467 |
|  | PPP2R5A |  | ✓ |  | ✓ |  | - |  | MIRT004602 |
|  | PAX3 |  | ✓ |  | ✓ |  | ✓ |  | MIRT004670 |
|  | SRSF9 |  | - |  | - |  | ✓ |  | MIRT005072 |
|  | TWF1 |  | ✓ |  | ✓ |  | ✓ |  | MIRT005089 |
|  | TWF2 |  | ✓ |  | ✓ |  | ✓ |  | MIRT005539 |
|  | CALM2 |  | - |  | - |  | ✓ |  | MIRT005904 |
|  | GATA6 |  | - |  | - |  | ✓ |  | MIRT005905 |
|  | SLC8A1 |  | ✓ |  | ✓ |  | - |  | MIRT006819 |
|  | EDN1 |  | ✓ |  | - |  | ✓ |  | MIRT006855 |
|  | PRKCE |  | ✓ |  | - |  | - |  | MIRT007205 |
|  | FABP3 |  | ✓ |  | ✓ |  | ✓ |  | MIRT007213 |
|  | SNAI2 |  | ✓ |  | ✓ |  | ✓ |  | MIRT007214 |
|  | SOX9 |  | ✓ |  | - |  | - |  | MIRT007222 |
|  | PGD |  | ✓ |  | ✓ |  | - |  | MIRT023542 |
|  | SRF |  | - |  | ✓ |  | - |  | MIRT023547 |
|  | IL11 |  | - |  | ✓ |  | - |  | MIRT023653 |
|  | YWHAZ |  | - |  | ✓ |  | ✓ |  | MIRT023741 |
|  | CCND1 |  | - |  | - |  | ✓ |  | MIRT023800 |
|  | MYOCD |  | - |  | ✓ |  | - |  | MIRT023927 |
|  | TKT |  | ✓ |  | ✓ |  | - |  | MIRT024031 |
|  | SP1 |  | ✓ |  | - |  | - |  | MIRT035536 |
|  | ETS1 |  | ✓ |  | ✓ |  | ✓ |  | MIRT054150 |
|  | FASN |  | - |  | ✓ |  | ✓ |  | MIRT054707 |
|  | PIK3CA |  | ✓ |  | ✓ |  | ✓ |  | MIRT054889 |
|  | TH |  | ✓ |  | - |  | - |  | MIRT437777 |
|  | MPL |  | ✓ |  | ✓ |  | ✓ |  | MIRT437822 |
|  | API5 |  | ✓ |  | ✓ |  | ✓ |  | MIRT437831 |
|  | SPRED1 |  | ✓ |  | ✓ |  | - |  | MIRT437883 |
|  | ASPH |  | ✓ |  | - |  | - |  | MIRT437918 |
|  | ND1 |  | ✓ |  | - |  | - |  | MIRT438458 |
|  | COX1 |  | ✓ |  | - |  | - |  | MIRT438459 |
|  | DUX4L9 |  | - |  | - |  | ✓ |  | MIRT438515 |
|  | FRS2 |  | ✓ |  | ✓ |  | ✓ |  | MIRT438811 |
|  | FZD7 |  | ✓ |  | ✓ |  | ✓ |  | MIRT438813 |
|  | AGO1 |  | ✓ |  | - |  | - |  | MIRT438862 |
|  | KRAS |  | ✓ |  | ✓ |  | ✓ |  | MIRT731362 |
|  | NAIP |  | ✓ |  | ✓ |  | ✓ |  | MIRT731787 |
|  | VEGFA |  | ✓ |  | ✓ |  | ✓ |  | MIRT731842 |
| miR-133a-3p | CDC42 |  | ✓ |  | ✓ |  | ✓ |  | MIRT000327 |
|  | HCN4 |  | ✓ |  | - |  | - |  | MIRT000329 |
|  | UCP2 |  | - |  | ✓ |  | - |  | MIRT000330 |
|  | KRT7 |  | - |  | - |  | ✓ |  | MIRT000331 |
|  | PNP |  | ✓ |  | ✓ |  | ✓ |  | MIRT006571 |
|  | CACNA1C |  | ✓ |  | ✓ |  | - |  | MIRT001203 |
|  | HCN2 |  | ✓ |  | ✓ |  | ✓ |  | MIRT001204 |
|  | CASP9 |  | ✓ |  | - |  | ✓ |  | MIRT001986 |
|  | KCNQ1 |  | ✓ |  | ✓ |  | ✓ |  | MIRT002925 |
|  | FSCN1 |  | ✓ |  | ✓ |  | ✓ |  | MIRT003542 |
|  | KCNH2 |  | ✓ |  | ✓ |  | ✓ |  | MIRT004831 |
|  | TAGLN2 |  | ✓ |  | ✓ |  | ✓ |  | MIRT005604 |
|  | LASP1 |  | ✓ |  | ✓ |  | ✓ |  | MIRT005813 |
|  | MSN |  | - |  | ✓ |  | ✓ |  | MIRT006680 |
|  | EGFR |  | ✓ |  | ✓ |  | ✓ |  | MIRT007032 |
|  | VKORC1 |  | ✓ |  | - |  | - |  | MIRT007088 |
|  | PRDM16 |  | ✓ |  | - |  | - |  | MIRT007383 |
|  | ARPC5 |  | ✓ |  | ✓ |  | ✓ |  | MIRT021705 |
|  | FTL |  | - |  | ✓ |  | - |  | MIRT021706 |
|  | EGFL7 |  | ✓ |  | - |  | - |  | MIRT021710 |
|  | VEGFA |  | ✓ |  | - |  | - |  | MIRT021711 |
|  | PIK3R2 |  | ✓ |  | - |  | - |  | MIRT021712 |
|  | RGS3 |  | ✓ |  | - |  | - |  | MIRT021713 |
|  | COL1A1 |  | ✓ |  | - |  | - |  | MIRT021714 |
|  | GSTP1 |  | ✓ |  | - |  | - |  | MIRT021715 |
|  | SP1 |  | ✓ |  | ✓ |  | - |  | MIRT035537 |
|  | BCL2L1 |  | ✓ |  | ✓ |  | ✓ |  | MIRT052647 |
|  | MCL1 |  | ✓ |  | ✓ |  | ✓ |  | MIRT052648 |
|  | RFFL |  | ✓ |  | ✓ |  | ✓ |  | MIRT053333 |
|  | IGF1R |  | ✓ |  | ✓ |  | ✓ |  | MIRT054310 |
|  | UBA2 |  | ✓ |  | ✓ |  | ✓ |  | MIRT081957 |
|  | MMP14 |  | ✓ |  | ✓ |  | ✓ |  | MIRT437400 |
|  | ANXA2 |  | ✓ |  | ✓ |  | ✓ |  | MIRT437953 |
|  | SNX30 |  | ✓ |  | ✓ |  | ✓ |  | MIRT437954 |
|  | SGMS2 |  | ✓ |  | ✓ |  | ✓ |  | MIRT437955 |
|  | DUX4L9 |  | - |  | - |  | ✓ |  | MIRT438514 |
|  | PDLIM5 |  | ✓ |  | ✓ |  | ✓ |  | MIRT438719 |
|  | IGF1 |  | ✓ |  | - |  | - |  | MIRT731340 |
|  | ZEB1 |  | ✓ |  | - |  | - |  | MIRT731591 |
| miR-206-3p | MET |  | ✓ |  | ✓ |  | ✓ |  | MIRT000185 |
|  | NOTCH3 |  | ✓ |  | ✓ |  | ✓ |  | MIRT000665 |
|  | ESR1 |  | ✓ |  | ✓ |  | ✓ |  | MIRT000700 |
|  | UTRN |  | ✓ |  | - |  | - |  | MIRT001788 |
|  | FSTL1 |  | ✓ |  | - |  | - |  | MIRT001789 |
|  | TAC1 |  | ✓ |  | - |  | - |  | MIRT003004 |
|  | PAX3 |  | ✓ |  | ✓ |  | ✓ |  | MIRT004671 |
|  | CCND2 |  | ✓ |  | - |  | - |  | MIRT007217 |
|  | GJA1 |  | - |  | ✓ |  | - |  | MIRT021080 |
|  | CDK4 |  | ✓ |  | - |  | - |  | MIRT052933 |
|  | ACTL6A |  | ✓ |  | ✓ |  | - |  | MIRT053344 |
|  | NR1H3 |  | ✓ |  | ✓ |  | ✓ |  | MIRT054618 |
|  | BCL2 |  | - |  | - |  | ✓ |  | MIRT199194 |
|  | NRP1 |  | ✓ |  | - |  | - |  | MIRT262367 |
|  | GPD2 |  | ✓ |  | ✓ |  | - |  | MIRT395575 |
|  | TKT |  | ✓ |  | ✓ |  | - |  | MIRT437465 |
|  | PGD |  | ✓ |  | ✓ |  | - |  | MIRT437466 |
|  | G6PD |  | ✓ |  | ✓ |  | - |  | MIRT437467 |
|  | VAMP2 |  | ✓ |  | - |  | - |  | MIRT437775 |
|  | SMARCB1 |  | - |  | - |  | ✓ |  | MIRT438222 |
|  | KRAS |  | ✓ |  | ✓ |  | ✓ |  | MIRT438292 |
|  | BDNF |  | - |  | - |  | ✓ |  | MIRT438353 |
|  | SFRP1 |  | - |  | - |  | ✓ |  | MIRT438354 |
|  | FRS2 |  | - |  | - |  | ✓ |  | MIRT438355 |
|  | IGF1R |  | - |  | - |  | ✓ |  | MIRT438356 |
|  | KLF4 |  | - |  | - |  | ✓ |  | MIRT438357 |
|  | HDAC4 |  | - |  | - |  | ✓ |  | MIRT438358 |
|  | STC2 |  | - |  | - |  | ✓ |  | MIRT438359 |
|  | CCND1 |  | ✓ |  | ✓ |  | - |  | MIRT438439 |
|  | DUX4L9 |  | - |  | - |  | ✓ |  | MIRT438512 |
|  | ANXA2 |  | ✓ |  | ✓ |  | ✓ |  | MIRT731150 |
| miR-383-5p | DIO1 |  | ✓ |  | ✓ |  | ✓ |  | MIRT006137 |
|  | IRF1 |  | ✓ |  | ✓ |  | ✓ |  | MIRT006535 |
|  | VEGFA |  | ✓ |  | - |  | - |  | MIRT004443 |
|  | IGF1R |  | ✓ |  | - |  | - |  | MIRT007246 |
|  | PRDX3 |  | ✓ |  | - |  | - |  | MIRT007305 |
|  | CCND1 |  | ✓ |  | ✓ |  | ✓ |  | MIRT054359 |
|  | PPP1R10 |  | ✓ |  | ✓ |  | ✓ |  | MIRT438194 |
