# Supplementary material for: Dietary supplementation of nucleotides and oligosaccharides in kittens reduces the expression of circulating miR-1-3p, miR-133a-3p, miR-206-3p and miR-383-5p
Source: Front Vet Sci. 2025 Nov 6;11:1382436. doi: 10.3389/fvets.2024.1382436 (PMC12632807; doi:10.3389/fvets.2024.1382436)
Supplement: Supplementary Table 4 — The miRNAs of interest target genes involved in immune signalling pathways. [file Table_4.docx]

**Supplementary Table 4 - The miRNAs of interest target genes involved in immune signalling pathways.**

The list of experimentally validated genes (identified in miRTarBase) targeted by the miRNAs of interest was used in the DAVID database to identify the pathways affected by the miRNAs. The results revealed that a number of target genes function in PI3K-AKT (20) and JAK-STAT (11) immune signalling pathways. False Discovery Rate (FDR).

|  |  |  |  |  |  |  |  |  |  |  |
| --- | --- | --- | --- | --- | --- | --- | --- | --- | --- | --- |
| **Pathways affected** |  | **Number of targeted genes** |  | **ID targeted genes** |  | ***P* value** |  | **Fold  Enrichment** |  | **FDR** |
|  |  |  |  |  |  |  |  |  |  |  |
| Pathways in cancer |  | 27 |  | BCL2; BCL2L1; CALM2; CALM3; CASP9; CCND1; CCND2; CDC42; CDK4; CEBPA; EDN1; EGFR; ESR1; ETS1; FN1; FZD7; GSTP1; IGF1; IGF1R; KRAS; MET; NOTCH3; PIK3CA; PIK3R2; PIM1; SP1; VEGFA |  | 1.00E-10 |  | 4.4 |  | 2.40E-08 |
| AGE-RAGE signalling pathway  in diabetic complications |  | 13 |  | BCL2; CCND1; CDC42; CDK4; COL1A1; EDN1; FN1; KRAS; PIK3CA; PIK3R2; PIM1; PRKCE; VEGFA |  | 1.10E-09 |  | 11.3 |  | 1.30E-07 |
| Proteoglycans in cancer |  | 16 |  | CCND1; CDC42; COL1A1; EGFR; ESR1; FN1; FRS2; FZD7; IGF1; IGF1R; KRAS; MET; MSN; PIK3CA; PIK3R2; VEGFA |  | 8.60E-09 |  | 6.8 |  | 5.80E-07 |
| Endocrine resistance |  | 12 |  | BCL2; CCND1; CDK4; EGFR; ESR1; IGF1; IGF1R; KRAS; NOTCH3; PIK3CA; PIK3R2; SP1 |  | 1.20E-08 |  | 10.6 |  | 5.80E-07 |
| PI3K-AKT signalling pathway |  | 20 |  | BCL2; BCL2L1; BDNF; CASP9; CCND1; CCND2; CDK4; COL1A1; EGFR; FN1; IGF1; IGF1R; KRAS; MCL1; MET; PIK3CA; PIK3R2; PPP2R5A; VEGFA |  | 1.20E-08 |  | 4.9 |  | 5.80E-07 |
| Prostate cancer |  | 11 |  | BCL2; CASP9; CCND1; EGFR; GSTP1; IGF1; IGF1R; KRAS; PIK3CA; PIK3R2; ZEB1 |  | 1.30E-07 |  | 9.8 |  | 4.90E-06 |
| Glioma |  | 10 |  | CALM2; CALM3; CCND1; CDK4; EGFR; IGF1; IGF1R; KRAS; PIK3CA; PIK3R2 |  | 1.50E-07 |  | 11.5 |  | 4.90E-06 |
| Pancreatic cancer |  | 10 |  | BCL2L1; CASP9; CCND1; CDC42; CDK4; EGFR; KRAS; PIK3CA; PIK3R2; VEGFA |  | 1.70E-07 |  | 11.4 |  | 4.90E-06 |
| EGFR tyrosine kinase inhibitor  resistance |  | 10 |  | BCL2; BCL2L1; EGFR; IGF1; IGF1R; KRAS; MET; PIK3CA; PIK3R2; VEGFA |  | 2.40E-07 |  | 11 |  | 6.20E-06 |
| MicroRNAs in cancer |  | 17 |  | BCL2; CCND1; CCND2; EGFR; FOXP1; FSCN1; HDAC4; KRAS; MCL1; MET; NOTCH3; PIK3CA; PIK3R2; PIM1; PRKCE; VEGFA; ZEB1 |  | 3.50E-07 |  | 4.7 |  | 8.20E-06 |
| Hepatocellular carcinoma |  | 13 |  | ACTL6A; BCL2L1; CCND1; CDK4; EGFR; FZD7; GSTP1; IGF1R; KRAS; MET; PIK3CA; PIK3R2; SMARCB1 |  | 3.90E-07 |  | 6.7 |  | 8.30E-06 |
| Breast cancer |  | 12 |  | CCND1; CDK4; EGFR; ESR1; FZD7; IGF1; IGF1R;  KRAS; NOTCH3; PIK3CA; PIK3R2; SP1 |  | 7.80E-07 |  | 7.1 |  | 1.50E-05 |
| Melanoma |  | 9 |  | CCND1; CDK4; EGFR; IGF1; IGF1R; KRAS; MET; PIK3CA; PIK3R2 |  | 1.40E-06 |  | 10.8 |  | 2.50E-05 |
| Ras signalling pathway |  | 14 |  | BCL2L1; BDNF; CALM2; CALM3; CDC42; EGFR; ETS1; IGF1; IGF1R; KRAS; MET; PIK3CA; PIK3R2; VEGFA |  | 2.00E-06 |  | 5.2 |  | 3.30E-05 |
| Focal adhesion |  | 13 |  | BCL2; CCND1; CCND2; CDC42; COL1A1; EGFR; FN1; IGF1; IGF1R; MET; PIK3CA; PIK3R2; VEGFA |  | 2.70E-06 |  | 5.6 |  | 4.10E-05 |
| Human papillomavirus infection | | 16 |  | ATP6V1B2; CCND1; CCND2; CDC42; CDK4; COL1A1; EGFR; FN1; FZD7; IRF1; KRAS; NOTCH3; PIK3CA; PIK3R2; PPP2R5A; VEGFA |  | 4.40E-06 |  | 4.2 |  | 6.30E-05 |
| Prolactin signalling pathway |  | 8 |  | CCND1; CCND2; ESR1; IRF1; KRAS; PIK3CA;  PIK3R2; TH |  | 1.30E-05 |  | 9.9 |  | 1.80E-04 |
| JAK-STAT signalling pathway |  | 11 |  | BCL2; BCL2L1; CCND1; CCND2; EGFR; IL11; MCL1; MPL; PIK3CA; PIK3R2; PIM1 |  | 1.40E-05 |  | 5.9 |  | 1.90E-04 |
| Non-small cell lung cancer |  | 8 |  | CASP9; CCND1; CDK4; EGFR; KRAS; MET;  PIK3CA; PIK3R2 |  | 1.60E-05 |  | 9.6 |  | 2.00E-04 |
| Measles |  | 10 |  | ADAR; BCL2; BCL2L1; CASP9; CCND1; CCND2; CDK4; MSN; PIK3CA; PIK3R2 |  | 2.70E-05 |  | 6.2 |  | 3.10E-04 |
|  |  |  |  |  |  |  |  |  |  |  |
|  |  |  |  |  |  |  |  |  |  |  |
